# Left-sided primary tumors are associated with favorable prognosis in patients with *KRAS* codon 12/13 wild-type metastatic colorectal cancer treated with cetuximab plus chemotherapy: an analysis of the AIO KRK-0104 trial

**DOI:** 10.1007/s00432-014-1678-3

**Published:** 2014-05-10

**Authors:** J. C. von Einem, V. Heinemann, L. Fischer von Weikersthal, U. Vehling-Kaiser, M. Stauch, H. G. Hass, T. Decker, S. Klein, S. Held, A. Jung, T. Kirchner, M. Haas, J. Holch, M. Michl, P. Aubele, S. Boeck, C. Schulz, C. Giessen, S. Stintzing, D. P. Modest

**Affiliations:** 1 Department of Medicine III, University Hospital Grosshadern, University of Munich, Munich, Germany; 2 Klinikum St. Marien, Amberg, Germany; 3 Onkologische Praxis Landshut, Landshut, Germany; 4 Onkologische Schwerpunktpraxis in Kronach, Kronach, Germany; 5 Paracelsus Klinik Scheidegg, Scheidegg, Germany; 6 Onkologie Ravensburg, Ravensburg, Germany; 7 Klinikum Bayreuth, Bayreuth, Germany; 8 ClinAssess, Gesellschaft für Klinische Forschung mbH, Leverkusen, Germany; 9 Department of Pathology, University of Munich, Munich, Germany

**Keywords:** Colorectal cancer, Primary tumor location, CAPIRI plus cetuximab, CAPOX plus cetuximab, *KRAS* mutation status

## Abstract

**Purpose:**

AIO KRK-0104 investigated first-line therapy of metastatic colorectal cancer (mCRC) with cetuximab, capecitabine and irinotecan versus cetuximab, capecitabine and oxaliplatin. This analysis investigated the impact of primary tumor location on outcome of patients.

**Patients and methods:**

Left-sided primary tumors were defined as tumors from rectum to left flexure, while tumors in the remaining colon were regarded right sided. Overall survival (OS), progression-free survival (PFS) and response rate were correlated with primary tumor location. A Cox regression model was used to evaluate interaction between primary tumor location and *KRAS* mutation.

**Results:**

Of 146 patients of the AIO KRK-0104 trial, 100 patients presented left-sided (of those 68 *KRAS* codon 12/13 wild-type) and 46 patients right-sided primary tumors (of those 27 *KRAS* codon 12/13 wild-type). Left-sided tumors were associated with significantly longer OS (*p* = 0.016, HR = 0.63) and PFS (*p* = 0.02, HR = 0.67) as compared to right-sided tumors. These effects were present in the *KRAS* codon 12/13 wild-type population (HR OS: 0.42; HR PFS: 0.54), while no impact of primary tumor location was evident in patients with *KRAS* codon 12/13 mutant tumors (HR OS: 1.3; HR PFS: 1.01). A significant interaction of *KRAS* status and primary tumor location concerning OS and PFS was observed.

**Conclusion:**

Our findings suggest that primary tumor location and *KRAS* codon 12/13 mutational status interact on the outcome of patients with mCRC receiving cetuximab-based first-line therapy. Left-sided primary tumor location might be a predictor of cetuximab efficacy.

## Introduction

The idea of personalized medicine was introduced to the treatment of metastatic colorectal cancer (mCRC) when *KRAS* codon 12/13 mutations were identified as negative predictors of anti-EGFR-antibody (EGFR-mAB) treatment. Consequently, only patients with *KRAS* codon 12/13 wild-type tumors were subjected to cetuximab or panitumumab treatment (Douillard et al. 2013; Huang et al. 2012; Modest et al. 2012; Douillard et al. 2010; Bokemeyer et al. 2011; Amado et al. 2008). This *KRAS* codon 12/13 wild-type population already excluded about 40 % of all patients and was associated with improved response rates (objective response rates, ORRs), progression-free survival (PFS) and overall survival (OS) in patients receiving EGFR-mABs. However, ORR in clinical trials investigating EGFR-based first-line regimens was usually <60 %, indicating that *KRAS* codon 12/13 wild-type alone was not a sufficient condition to predict response (Douillard et al. 2013; Modest et al. 2012; Van Cutsem et al. 2011; De Roock et al. 2010; Stintzing et al. 2009). The identification of additional negative predictors such as *KRAS* exon 3/4 and *NRAS* exon 2–4 mutations created a new target population for EGFR-mABs: patients with RAS wild-type tumors. This population comprises about 50 % of all patients with mCRC with a benefit in median OS following EGFR-targeted first-line therapy of 5–7 months (Douillard et al. 2013; Stintzing et al. 2009).

Taking into account that even RAS wild-type tumors potentially do not define the perfect marker for response to EGFR-mABs, additional biomarkers are needed. This question was recently addressed by retrospective evaluations of patients receiving cetuximab treatment in further treatment lines. The efficacy of cetuximab was determined to be modulated by the location of the primary tumor (Missiaglia et al. 2013; Brule et al. 2013). Due to this initial evidence, the question was raised whether the location of the primary tumor in colorectal cancer can serve as a prognostic marker and potentially as a predictive marker for treatment with EGFR-mABs. To our knowledge, the effect of primary tumor location on outcome has not been shown in a mCRC study population receiving first-line treatment with cetuximab.

The AIO KRK-0104 trial randomized patients to CAPIRI plus cetuximab or CAPOX plus cetuximab. With reference to this design, we hypothesized that primary tumor location of the left colon might have a favorable prognostic effect in patients with *KRAS* wild-type tumors, but not in patients with *KRAS* mutant tumors.

## Methods

### Study design

Data for this analysis were obtained from the AIO KRK-0104 trial. This study was a randomized, multicenter phase II trial to investigate the efficacy of cetuximab plus CAPIRI versus cetuximab plus CAPOX as first-line chemotherapy in patients with mCRC and recruited patients from 2004 to 2006. The primary analysis and the molecular subgroups analysis have been published elsewhere (Modest et al. 2012; Moosmann et al. 2011). Primary endpoint of the AIO KRK-0104 study was ORR. This investigation refers to the population of 146 patients with central assessment of *KRAS/BRAF* mutations as published before (Modest et al. 2012).

### Definition of right-sided versus left-sided tumors

The primary tumor location was defined in the study reports and was extracted from the central database. Tumors located in rectum, sigma, descending colon and the left flexure were defined as left-sided tumors. All tumors from cecum to the distal part of the transverse colon were categorized as right-sided tumors.

### Treatment schedule

In both arms, cetuximab was given at an initial dose of 400 mg/m^2^ as a 120-min infusion, followed by weekly infusions of 250 mg/m^2^ over 60 min. Patients in arm A received chemotherapy with CAPIRI (i.e., oral capecitabine 800 mg/m^2^ twice daily on days 1 through 14, followed by a 1-week rest period plus irinotecan 200 mg/m^2^ as a 30-min intravenous infusion on day 1). In patients older than 65 years, doses were further reduced by 20 %. Patients in arm B received chemotherapy with CAPOX (i.e., capecitabine 1,000 mg/m^2^ twice daily on days 1 through 14, followed by a 1-week rest period plus oxaliplatin 130 mg/m^2^ as a 120-min intravenous infusion on day 1). Treatment cycles were repeated every 3 weeks until disease progression or unacceptable toxicity (Moosmann et al. 2011).

### Patients

The patient population of the AIO KRK-0104 trial was described in recent reports (Modest et al. 2012; Moosmann et al. 2011). Patients with *BRAF* mutant tumors were analyzed as *KRAS* wild-type tumors. One patient presenting a tumor with *BRAF* and *KRAS* mutation was regarded as *KRAS* mutant in this analysis. In two patients, two primary tumors were located in the left-sided colon (sigma and rectum; descendent colon and sigma); these cases were analyzed as left-sided colorectal cancer. In another patient, one primary tumor was located in the right part of the colon (cecum), while another primary tumor was observed at the left side (rectum); this case was classified as right-sided colorectal cancer.

### Endpoints

The present investigation was performed as an exploratory analysis using response rates (ORR = complete and partial remission), PFS and OS as parameters for outcome in patients with tumors of right-sided and left-sided origin. Tumor assessment was performed every two cycles (6 weeks). A final update on overall survival was conducted in 2011, and the statistical analysis plan was published in detail (Modest et al. 2012; Moosmann et al. 2011).

### Statistical analysis

In this retrospective, exploratory investigation, OS and PFS were stratified by primary tumor location and were estimated using the Kaplan–Meier method. Possible differences were evaluated by log-rank test and Cox regression analysis. A Cox regression model was used to evaluate interaction between primary tumor location and *KRAS* mutation as explanatory variables. *χ* ^2^ tests compared response rates. A *p* value <0.05 was regarded significant. For interaction test, a *p* value <0.10 was regarded significant. SPSS PASW 21.0 (SPSS Inc., Chicago, Illinois) and SAS 9.2 (SAS Institute Inc., Cary, NC, USA) were used for statistical analysis.

### *KRAS* mutation detection

*KRAS/BRAF* testing was performed in a German reference laboratory for *KRAS* analysis (University of Munich, Department of Pathology) as described before (Modest et al. 2012; Moosmann et al. 2011).

## Results

### Study population and tumor characteristics

In all 146 patients of the pathological analysis-set, the primary tumor location was assessable. Out of the full population, 100 patients presented with left-sided tumors, whereas 46 patients presented primary right-sided tumors. In detail, tumors were located in rectum (*n* = 49), sigma (*n* = 40), descending colon (*n* = 7), left flexure (*n* = 2), transverse colon (*n* = 11), ascending colon (*n* = 18), cecum (*n* = 16) and double primary location (*n* = 3). Out of the 100 patients presenting left-sided colorectal tumors, 68 tumors presented *KRAS* codon 12/13 wild-type tumors, and 32 tumors had *KRAS* codon 12/13 mutations. Out of 46 tumors of right-sided origin, 27 tumors were diagnosed with *KRAS* codon 12/13 wild-type status, while 19 patients presented a *KRAS* codon 12/13 mutant tumor. Distribution of patients with left-sided versus right-sided tumors to the treatment arms of the AIO KRK-0104 trial (CAPIRI plus cetuximab/CAPOX plus cetuximab) was comparable (52/48 % vs. 50/50 %; Table 1).

**Table 1:** Baseline characteristics of patients and tumors

	Patients with left-sided mCRC		Patients with right-sided mCRC		*p* value
	*n*	%	*n*	%	
Patients	100	68	46	32	
*Age*					
Median	63		61		0.50
Range	32–77		47–74		
*Sex*					
Female	23	23	18	39	0.05
Male	77	77	28	61	
*Performance status (Karnofsky)*					
100 + 90	73	73	32	70	0.55
80 + 70	25	25	14	30	
Not reported	2	2	0	0	
*Prior therapy*					
Chemotherapy	21	21	5	11	0.17
Radiotherapy	12	12	1	2	0.06
*Disease sites*					
Liver	84	84	42	91	0.31
Lung	32	32	19	41	0.35
Lymph node	30	30	20	43	0.13
Peritoneum	12	12	3	7	0.39
*Treatment arm*					
CAPIRI plus cetuximab	52	52	23	50	0.86
CAPOX plus cetuximab	48	48	23	50	
*Tumor mutation status*					
*KRAS* codon 12/13 wild-type	68	68	27	59	0.35
*KRAS* codon 12/13 mutant	32	32	19	41	
*BRAF* V600E mutant	6	6	11	24	0.004

*p* values: Chi-square test/Fisher’s exact test except for age: Mann–Whitney *U* test

### Baseline patient characteristics

Baseline patient characteristics are shown in Table 1. No major imbalances associated with primary tumor location in the left or right part of the colon were present in our cohort. However, a trend toward more female patients was observed in the group of right-sided tumors when compared to the group of patients with left-sided primary tumors (39 % vs. 23 %, *p* = 0.05; Table 1).

### Effect of primary tumor location on overall survival (OS)

**Fig. 1:**
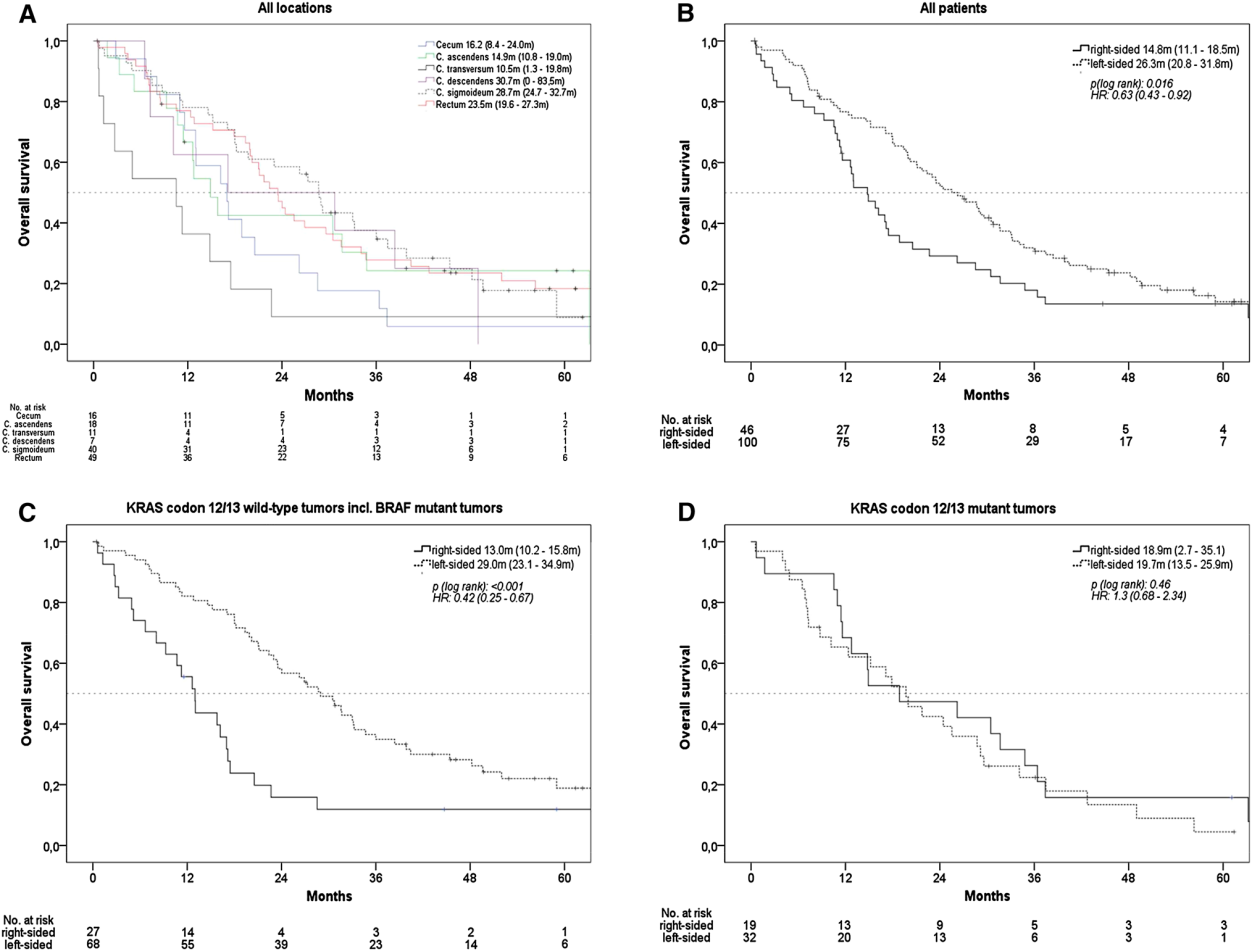
Overall survival (OS), **a** according to exact primary tumor location, patients with double primary tumors and with primary tumor of left flexure were excluded due to sample size, **b** according to right-sided versus left-sided mCRC, **c** patients with KRAS codon 12/13 wild-type tumors, **d** patients with KRAS codon 12/13 mutant tumors

The whole study population reached a median OS of 21.1 months. Survival times by exact tumor locations are shown in Fig. 1a. If analyzed as right vs. left colon, median OS of patients with right-sided tumor was 14.8 months, while median OS in patients with left-sided tumor was 26.3 months (*p* = 0.016, HR = 0.63), (Fig. 1b). In patients with *KRAS* codon 12/13 wild-type tumors, median OS was 13.0 months in patients with right-sided versus 29.0 months in patients with left-sided mCRC (*p* < 0.001, HR: 0.42). The effect of primary tumor location on OS in patients with *BRAF* V600E mutant tumors seemed consistent with the observation in the *KRAS* codon 12/13 wild-type cohort. In patients with *KRAS* codon 12/13 mutant tumors, no significant difference was present when OS of patients with right-sided and left-sided mCRC was compared (Figs. 1c–d and 3a).

### Effect of primary tumor location on progression-free survival (PFS)

**Fig. 2:**
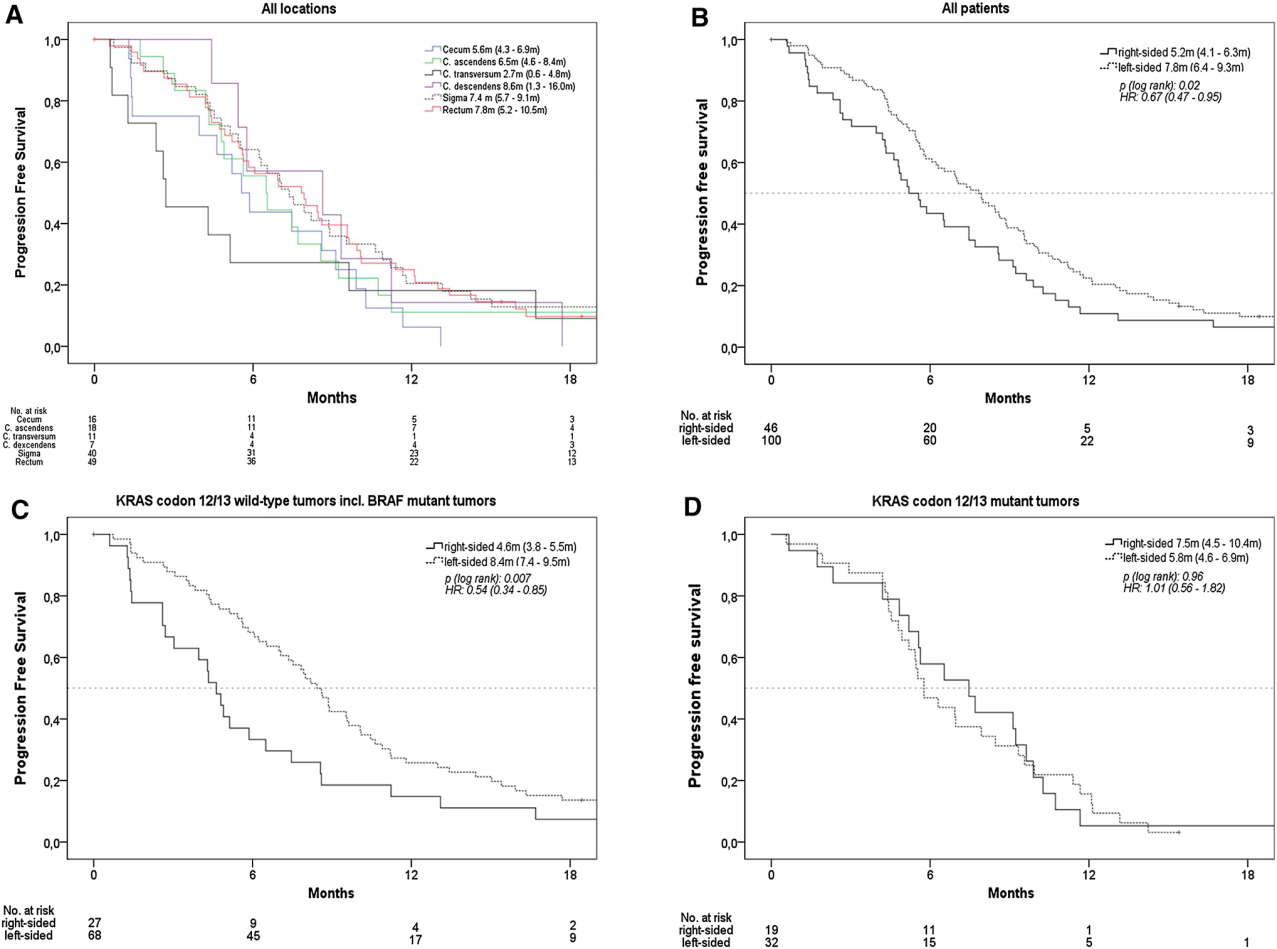
Progression-free survival (PFS), **a** according to exact primary tumor location patients with double primary tumors and with primary tumor of left flexure were excluded due to sample size, **b** according to right-sided versus left-sided mCRC, **c** patients with KRAS codon 12/13 wild-type tumors, **d** patients with KRAS codon 12/13 mutant tumors

**Fig. 3:**
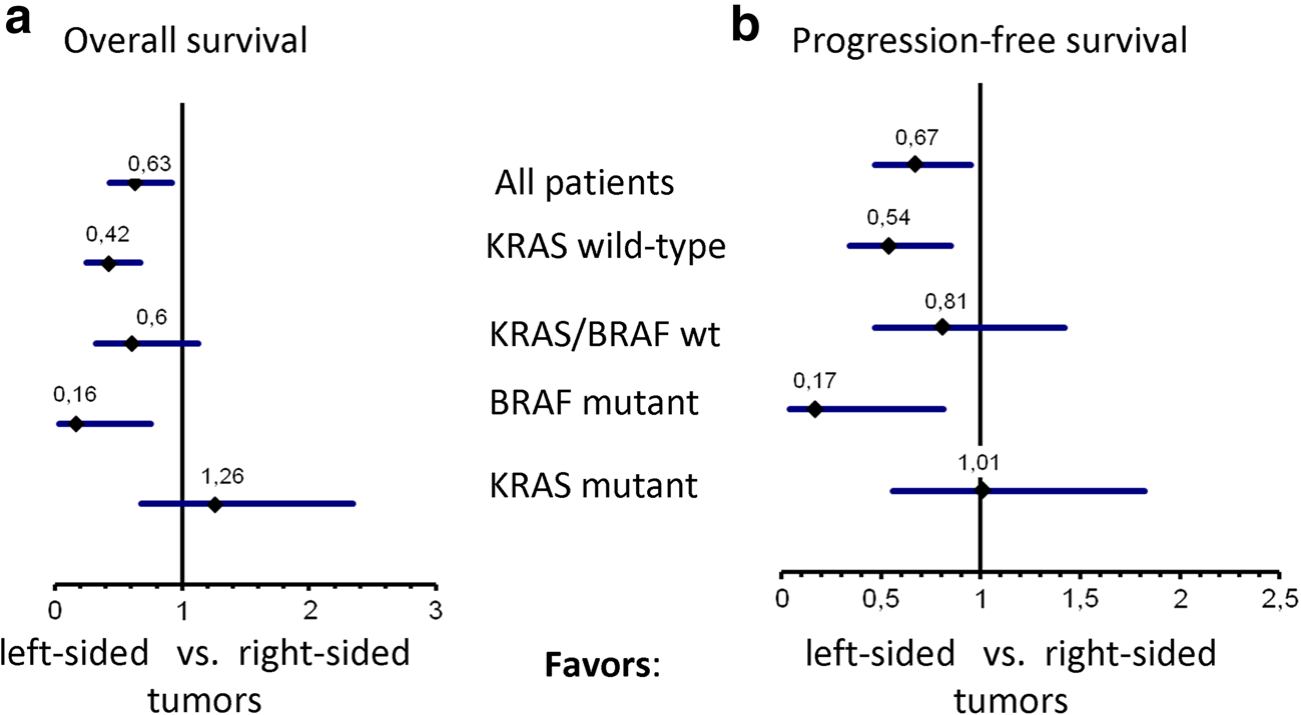
Hazard ratios of molecular subgroups, error bars indicating the 95 % confidence interval. **a** Overall survival, **b** progression-free survival

Median PFS in all patients was 7.0 months. PFS by exact tumor location is shown in Fig. 2a. In patients with right-sided tumors, median PFS was 5.2 months, and in patients with left-sided-tumors, it was 7.8 months (*p* = 0.02, HR = 0.67). If *KRAS* status was taken into account, in accordance to OS, a significant difference in PFS associated with primary tumor location was only evident in patients with *KRAS* codon 12/13 wild-type tumors (4.6 vs. 8.4 months, *p* = 0.007, HR = 0.54), but not in patients presenting a mutation in these loci. In patients with *BRAF* mutant mCRC, the effect of primary tumor location seemed again consistent with the effects observed in patients with *KRAS* codon 12/13 wild-type tumors (Figs. 2b–d and 3b).

### Interaction of *KRAS* codon 12/13 mutation and primary tumor location

Cox regression models with hazard ratios for primary tumor location, *KRAS* mutation and interaction respectively as explanatory variables were analyzed. For OS, the hazard ratio of primary tumor location was 0.160 in favor of left-sided (*p* = 0.002, 95 % CI 0.050–0.511), 0.640 in favor of wild-type (*p* = 0.159, 95 % CI 0.344–1.191) for *KRAS* mutation, and 0.372 (*p* = 0.013, 95 % CI 0.171–0.810) for interaction between primary tumor location and *KRAS* mutation. For PFS, the hazard ratio of primary tumor location was 0.252 in favor of left-sided tumors (*p* = 0.013, 95 % CI 0.085–0.745), 0.780 in favor of wild-type (*p* = 0.411, 95 % CI 0.432–1.140) for *KRAS* mutation, and 0.487 (*p* = 0.056, 95 % CI 0.233–1.017) for interaction between primary tumor location and *KRAS* mutation.

### Influence of *BRAF* V600E mutations in this study

In our cohort, BRAF mutation was more frequent in right-sided compared with left-sided primary tumors (24 vs. 6 %). After removing *BRAF* V600E mutant tumors from the *KRAS* codon 12/13 wild-type cohort, median PFS of patients with *KRAS/BRAF* wild-type tumors (*n* = 79) was 5.9 months in patients with right-sided versus 8.2 months with left-sided tumors (*p* = 0.47, HR = 0.81). Median OS was 16.2 months in patients with right-sided compared with 27.3 months in patients with left-sided tumors (*p* = 0.11, HR = 0.60; Fig. 3a, b).

### Effect of primary tumor location on response rate

Response rates were analyzed based on non-missing data and did not show significant differences. ORR was 58 % in left-sided and 53 % in right-sided tumors (*p* = 0.70) in the whole study population. In *KRAS* codon 12/13 wild-type tumors, right-sided tumors were associated with a lower ORR when compared to left-sided tumors (43 % vs. 64 %), and this difference did not reach significance (*p* = 0.12). When comparing response rates in patients with *KRAS* codon 12/13 mutant mCRC, ORR was 65 % in right-sided and 45 % in left-sided tumors (*p* = 0.23; Table 2).

**Table 2:** Response to treatment

Parameter	L-mCRC	R-mCRC	L-mCRC KRAS wt	R-mCRC KRAS wt	L-mCRC KRAS mut	R-mCRC KRAS mut
No. of patients	100	46	68	27	32	19
ORR evaluable (no. of pts)	85	38	56	21	29	17
n.a. (no. of pts)	15	8	12	6	3	2
ORR % (95 % CI)	58 (47–68)	53 (37–68)	64 (51–77)	43 (24–65)	45 (27–65)	65 (40–86)
*p* value	0.70		0.12		0.23	

*R-mCRC* patients with right-sided mCRC, *L-mCRC* patients with left-sided mCRC, *wt* wild-type, *mut* mutant; *ORR* (CR + PR) overall response rate, *CI* confidence interval, *n.a* not assessable due to any reason. *p* value: Fisher’s exact test. ORR calculation based on non-missing data (patients evaluable for response)

## Discussion

Personalized treatment of mCRC patients is entering daily routine in clinical practice. The more tumor sub-classifications based on molecular markers are defined, the higher the chance is to identify positive and negative predictors and consequently to specify different strategies of therapy. Clinical data have proven that mutant RAS genes are negative predictive biomarkers and that patients with a *KRAS/NRAS* mutation do not benefit from an EGFR-mAB-based therapy (De Roock et al. 2010; Peeters et al. 2013a, b; Andre et al. 2013). Therefore, mutations of *KRAS* and *NRAS* represent an established negative predictor of EGFR-mAB efficacy. The role of *BRAF* in first-line treatment of mCRC is described as a negative prognostic marker, but not as a predictive marker in terms of EGFR-mAB therapy (Douillard et al. 2013). Recently, it has been suggested that, in addition to RAS mutations, the primary tumor location might play a crucial role for efficacy of EGFR-mABs (Douillard et al. 2013; Missiaglia et al. 2013).

For this reason, we hypothesized that in the AIO KRK-0104 trial primary tumor location in the left colon might have a favorable prognostic effect in patients with *KRAS* wild-type tumors, but not in patients with *KRAS* mutant tumors when receiving cetuximab-based first-line therapy. In fact, OS and PFS differed significantly when comparing left- to right-sided tumors. This effect was driven by patients with *KRAS* codon 12/13 wild-type tumors and seemed also present in those patients that presented with *KRAS* codon 12/13 wild-type but *BRAF* V600E mutant tumors. By contrast, in patients with *KRAS* codon 12/13 mutant tumors, the primary tumor location was not associated with significant differences in terms of OS or PFS. This interaction of *KRAS* mutation and primary tumor location was found to be significant for both OS and PFS. No significant impact of primary tumor location on response rates was observed in this study. Taking the difference in patients with *KRAS* wild-type tumors into account (64 % vs. 43 % in patients with left-sided vs right-sided primary tumor), this could be interpreted as a consequence of missing sample size for this endpoint.

Our results are supported by a recent analysis of the NCIC CTG CO.17 trial (that investigated cetuximab plus best supportive care (BSC) versus BSC alone), which reported less striking cetuximab-induced effects in patients with *KRAS* codon 12/13 wild-type, right-sided tumors as compared with patients bearing a left-sided tumor (Brule et al. 2013). A similar observation was reported by Missiaglia and colleagues who observed a longer PFS in refractory patients that received cetuximab treatment if the primary tumor was left-sided as compared to right-sided tumors (Missiaglia et al. 2013).

As described above, we grouped patients in left-sided versus right-sided tumors, which included tumors from cecum to the distal part of the transverse colon. This distinction corresponds to the midgut versus hindgut definition and is modified counting the total colon transversum as right-sided colon. Nevertheless, the strict separation of different tumor locations is questioned by the “continuum hypothesis,” which postulates that molecular features of the tumor gradually change along bowel subsides, rather than change abruptly at splenic flexure (Yamauchi et al. 2012a, b). As shown in Figs. 1a and 2a, a trend toward specific OS and PFS could possibly be derived from the exact primary tumor location of the colon, but possibly not according to the physiological course of the colon. Clearly, our data concerning this issue are limited by sample size.

As samples from the AIO KRK-0104 trial were only tested for *KRAS* exon 2 codon 12/13 mutations, but not for *KRAS* exon 3, 4 or *NRAS* mutations, our data might contain a bias of approximately 10 % hidden mutations that we cannot identify due to lacking tumor material. In our cohort, the impact of left- versus right-sided tumors was specifically strong in *BRAF* mutant tumors. This finding might be explainable by sample size and MSI/MSS status that is unknown for the tumors of AIO KRK-0104 trial cohort. It might have been suspected that the whole side effect might be influenced by *BRAF/*MSI/MSS status, since *BRAF* mutations are known to be more frequent in right-sided colorectal cancer (Pai et al. 2012; Popovici et al. 2013). However, even after excluding *BRAF* mutant tumors from the *KRAS* codon 12/13 wild-type cohort, the strong prognostic effect of left-sided primary tumors seemed still present (hazard ratio for OS: 0.60). It might be concluded that in mCRC *BRAF* mutation interacts in the left- versus right-sided tumor story, but is not the only reason for the observed differences.

Our data are limited by several aspects. As discussed above, we distinguished between *KRAS* mutant and non-mutant only and did not take other RAS mutations into account. Furthermore, this study only consists of a rather small population that might lead to biases, especially in the *KRAS* mutant cohort. Furthermore, treatment differences between oxaliplatin- and irinotecan-treated patients could not be excluded.

In conclusion, our data correspond favorably with other publications investigating EGFR-mAB use and primary tumor location in mCRC. The interaction of primary tumor location and *KRAS* mutations suggests that primary tumor location might be an additional biomarker for EGFR-mABs. Corresponding pathological findings to explain this phenomenon are still lacking and could be more complex than RAS mutations (Missiaglia et al. 2013; Maus et al.^ 2013^). Data from randomized phase III trials such as CRYSTAL, PRIME and FIRE 3 are necessary to draw definite conclusions concerning the restriction of EGFR-mAbs to patients with RAS wild-type left-sided mCRC.
